# Juvenile handling rescues autism-related effects of prenatal exposure to valproic acid

**DOI:** 10.1038/s41598-022-11269-7

**Published:** 2022-05-03

**Authors:** Araceli Seiffe, Mauro Federico Ramírez, Lucas Sempé, Amaicha Mara Depino

**Affiliations:** 1 Departamento de Fisiología, Biología Molecular y Celular, Facultad de Ciencias Exactas y Naturales, Universidad de Buenos Aires, C1428EHA Buenos Aires, Argentina; 2 Instituto de Fisiología, Biología Molecular y Neurociencias (IFIBYNE), CONICET-Universidad de Buenos Aires, Int. Guiraldes 2160, Ciudad Universitaria, Edificio IFIBYNE, C1428EHA Buenos Aires, Argentina; 3 Institute for Global Health and Development, Queen Margaret University Edinburgh, Edinburgh, UK; 4 Departamento de Biodiversidad y Biología Experimental, Facultad de Ciencias Exactas y Naturales, Universidad de Buenos Aires, Buenos Aires, Argentina

**Keywords:** Autism spectrum disorders, Social behaviour, Stress and resilience

## Abstract

Environmental factors acting on young animals affect neurodevelopmental trajectories and impact adult brain function and behavior. Psychiatric disorders may be caused or worsen by environmental factors, but early interventions can improve performance. Understanding the possible mechanisms acting upon the developing brain could help identify etiological factors of psychiatric disorders and enable advancement of effective therapies. Research has focused on the long-lasting effects of environmental factors acting during the perinatal period, therefore little is known about the impact of these factors at later ages when neurodevelopmental pathologies such as autism spectrum disorder (ASD) are usually diagnosed. Here we show that handling mice during the juvenile period can rescue a range of behavioral and cellular effects of prenatal valproic acid (VPA) exposure. VPA-exposed animals show reduced sociability and increased repetitive behaviors, along with other autism-related endophenotypes such as increased immobility in the forced swim test and increased neuronal activity in the piriform cortex (Pir). Our results demonstrate that briefly handling mice every other day between postnatal days 22 and 34 can largely rescue these phenotypes. This effect can also be observed when animals are analyzed across tests using an “autism” factor, which also discriminates between animals with high and low Pir neuron activity. Thus, we identified a juvenile developmental window when environmental factors can determine adult autism-related behavior. In addition, our results have broader implications on behavioral neuroscience, as they highlight the importance of adequate experimental design and control of behavioral experiments involving treating or testing young animals.

## Introduction

Autism spectrum disorder (ASD) is characterized by reduced sociability, diminished communicative skills, and repetitive behaviors^1^. Symptoms are present in the early developmental period, although they may not manifest fully until social demands exceed the limited capacities^1^. The prevalence for ASD in the total population is estimated to be as high as 1 in every 38 children^2,3^, and reports show that it keeps rising^4^.

ASD is a neurodevelopmental condition of heterogeneous etiology^5^. Initially, it was considered the most heritable of all neurodevelopmental disorders mainly because the concordance rates were largely different between monozygotic and dizygotic twins. However, subsequent evidence showed that environmental factors common to twins can explain about 55% of the liability to autism, henceforth conditioning the influence of genetic factors^6^. In fact, only about 10–20% of individuals with ASD have an identified genetic etiology^7^ and there is a robust body of compelling evidence that environmental and epigenetic factors may have a role in ASD susceptibility^8^. In addition, most research examining the genetic and environmental contributions to the etiology of ASD has largely analyzed factors in isolation, rather than considering the role of gene–environment interactions through processes such as epigenetic dysregulation, an approach that may shed light on novel etiological mechanisms involved in ASD^5^. Indeed, environment can affect multiple aspects of ASD: An early harmful environment can *cause* ASD or *mediate* between a genetic predisposition and the actual manifestation of the disorder. Conversely, a rich and supportive environment can *moderate* the severity of ASD and even *protect* against the risk of developing ASD symptoms.

Prenatal exposure to anticonvulsants shows the strongest clinical evidence among causal environmental factors that increase risk of developing ASD. In particular, children born to mothers treated with valproic acid (VPA) during pregnancy suffer from somatic malformations and behavioral alterations, presenting remarkable autistic features^9^. Following that evidence, we^10,11^ and others^12–14^ have shown that prenatal exposure of mice to 600 mg/kg VPA at gestational day (GD) 12.5 results in different autism-related behaviors. These alterations include reduced sociability, increased repetitive behaviors and depression-related behaviors^10,11,14^. Moreover, other endophenotypes are also observed in the offspring of dams injected with VPA, such as increased neuroinflammation and an altered stress response^11,15^. These parameters are also observed in individuals with ASD^16^. Moreover, VPA affects the sociability of male offspring but spares their female littermates^17,18^, showing a sex bias also observed is ASD, where boys are three to four times more likely to be affected than girls^19–21^. These previous studies give the VPA model high validity and places it in a privileged situation to explore possible ASD therapies.

Among protective and moderating environmental factors, early social enrichment can profoundly impact the development of children at risk for ASD. Indeed, clinical studies indicate that strategies of social involvement may promote early social communicative skills in affected children^22,23^. Although the specific mechanisms of action are unknown, early social enrichment can alter gene expression, brain development, and behavioral outcome in ASD^23^. In line with these clinical observations, we previously established that weaning VPA mice with control mice at postnatal day 21 (PD21) rescues the reduction in sociability observed in adult VPA mice reared with other VPA peers^10^. These results indicate that early social enrichment can specifically rescue social deficits in VPA mice and the existence of a critical period between PD21 and PD60, when future levels of sociability can be programmed by environmental factors such as daily interactions with peers. In this line, we previously observed that male mice prenatally exposed to VPA that later received intraperitoneal injections between PD22 and PD34 showed normal sociability levels in adulthood, reverting the social deficit typically observed in these animals (unpublished data). We then reasoned that this effect could be due to the handling of juvenile animals necessary for the injections, a procedure that usually takes around 3 min.

“Handling” refers to the interaction of the experimenter with the animal, and it is usually performed to habituate rodents to the experimental situation and reduce their stress. However, handling is also known as one of the first rodent models applied to investigate the long-term effects of early-life experiences on brain development^24^. Indeed, studying the effects of postnatal handling on brain development, helped understanding how subtle changes in the early environment can alter the typical neurodevelopmental trajectories^25^. Moreover, long-lasting effects indicate that, although experimental handling of rodents does not mimic a natural environmental stimulus, biologically relevant pathways are activated by the procedure.

Since multiple outcomes of early handling have been reported and the effects of post-weaning stimulation on sociability remain unknown, we aimed to investigate how subjecting animals to juvenile handling (JH) between PD22 and PD34 affects the outcome of the VPA mouse model of autism. In particular, we evaluated the effect of JH on behaviors relevant to ASD that are affected by prenatal exposure to VPA: sociability (social interaction test), repetitive behaviors (self-grooming and Y maze alternation), and depression-related behaviors (tail suspension and forced swim tests). In addition, we previously identified the piriform cortex (Pir) as a structure affected by prenatal VPA exposure and social enrichment^10^. We evaluated the effects of JH on basal neuronal activity in the Pir by analyzing the expression of the early response transcription factor cFos.

## Results

### Handling mice between PD22 and PD34 rescues the sociability alterations observed after prenatal exposure to VPA

**Figure 1:**
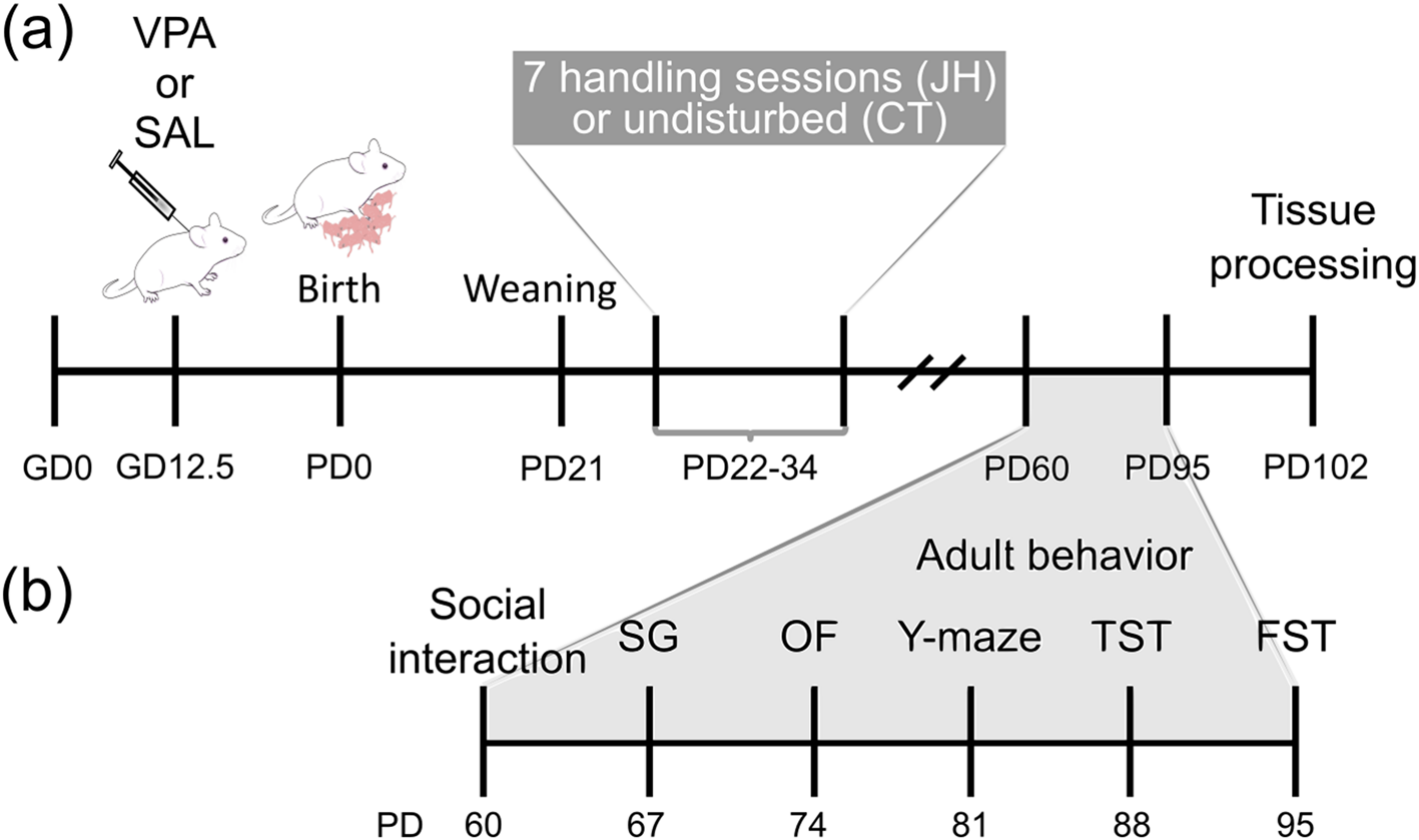
Experimental design. (**a**) Animals were prenatally exposed to valproic acid (VPA) or saline (SAL), weaned at postnatal day (PD) 21 and then subjected to juvenile handling (JH) or left undisturbed (CT). (**b**) In adulthood, animals were evaluated in the social interaction test, self-grooming test (SG), open field test (OF), Y-maze test, tail suspension test (TST) and forced swim test (FST).

Male offspring born to mothers injected with valproic acid (VPA) or saline (SAL) at gestational day (GD) 12.5, were subsequently subjected to 7 handling sessions of 3 min each between postnatal day (PD) 22 and PD34 (juvenile handling, JH) or left undisturbed (control, CT; Fig. 1a). Animals were then allowed to reach adulthood (PD60) and then we evaluated their autism-related behaviors across a series of behavioral tests (Fig. 1b). We weighed the animals after each behavioral test and observed an effect of prenatal treatment [F (1, 49.02) = 5.173, p = 0.027], with VPA-exposed mice being lighter (Supplemental Fig. 1), an effect previously reported^15,26^. Juvenile handling had no effect on adult body weight [F (1, 49.02) = 0.407, p = 0.527].

**Figure 2:**
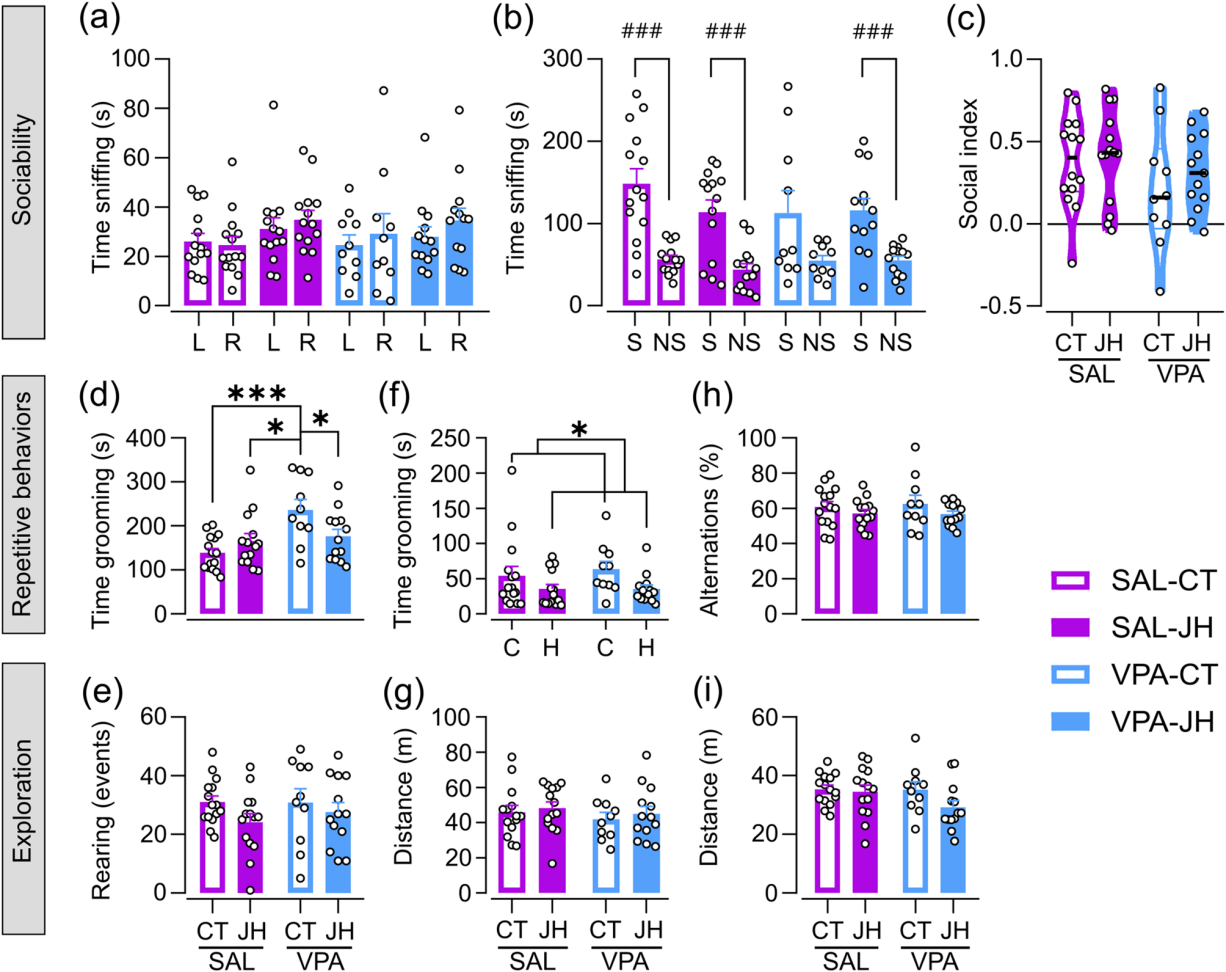
Effect of prenatal VPA exposure and juvenile handling (JH) on adult mice sociability, repetitive behavior, and exploration. (**a–c**) Three-chamber test: (**a**) Animals show no preference for the left (L) or the right (R) cylinder in the habituation phase. (**b**) VPA-CT animals lack the preference for the social stimulus (S) over the nonsocial cylinder (NS) observed in control groups. JH results in normalization of VPA mice sociability. Paired Student’s *t* test, ^###^p < 0.001. (**c**) VPA-CT animals show a tendency of reduced social index. (**d,e**) Self-grooming test: (**d**) VPA-CT animals spend more time in grooming than all other groups. LME model followed by Tukey’s post hoc comparisons, *p < 0.05, ***p < 0.001. (**e**) All animals perform similar rearing events. (**f,g**) Open field test: (**f**) Animals handled post-weaning spend less time grooming in the open field. GLMM model, *p < 0.05. (**g**) Mice walk a similar distance in the arena. (**h,i**) Y maze test: (**h**) No differences among groups were observed in the percentage of alternations in the Y maze. (**i**) All animals explore the Y maze similarly. Data are shown as individual values (dots) and mean + s.e.m., except for the violin plot in (**c**). N_SAL-CT_ = 14–15, N_SAL-JH_ = 14, N_VPA-CT_ = 10, N_VPA-JH_ = 13.

We first evaluated sociability using the three-chamber social interaction test. During the habituation session, all mice showed similar levels of investigation [prenatal treatment: F (1, 22.763) < 0.001, p = 0.987; juvenile treatment: F (1, 25.145) = 3.188, p = 0.086], and we observed no preference for either side [Paired Student’s *t* test, SAL-CT: t (13) = 0.323, p = 0.751; SAL-JH: t (13) =  − 1.215, p = 0.246; VPA-CT: t (9) =  − 0.518, p = 0.617; VPA-JH: t (12) =  − 1.127, p = 0.282; Fig. 2a]. In the social phase, as expected, control animals preferred to explore the social cylinder [Paired Student’s *t* test, SAL-CT: t (13) = 4.482, p < 0.001; SAL-JH: t (13) = 4.598, p < 0.001; Fig. 2b] while VPA-CT animals showed no preference for neither side [VPA-CT: t (9) = 1.957, p = 0.082] as previously reported^17^. Interestingly, VPA-JH mice preferred to investigate the stimulus mouse [t (12) = 4.263, p = 0.001], showing that the handling procedure is sufficient to rescue the alteration in sociability observed in VPA animals. Finally, we observed no effects of prenatal and juvenile treatments in the social index [prenatal treatment: F (1, 24.657) = 2.077, p = 0.162; juvenile treatment: F (1, 25.156) = 1.174, p = 0.288; Fig. 2c].

### Juvenile handling rescues VPA-induced alterations in repetitive behaviors

Next, we evaluated repetitive behaviors via the self-grooming and Y-maze tests, and by quantifying the time spent in self-grooming in the open field test. In the self-grooming test, we found an interaction effect in the time that mice spent grooming [F (1, 24.069) = 13.3460, p = 0.001], and post hoc analyses evidenced that the VPA-CT group spent more time in grooming than the other groups (Fig. 2d). No effect of prenatal treatment [GLMM, χ^2^ (1, 46) = 0.282, p = 0.595] was observed in grooming in the open field, but handled mice groomed less in this test [juvenile treatment: χ^2^ (1, 46) = 8.022, p = 0.004; Fig. 2f]. Finally, we observed no differences among groups in the percentage of alternations in the Y maze [prenatal treatment: F (1, 27.584) = 0.003, p = 0.953; juvenile treatment: F (1, 29.736) = 3.243, p = 0.082; Fig. 2h]. These results show that JH can rescue VPA-induced alterations in repetitive behaviors and this procedure can also have an effect on grooming in a novel environment in control animals.

We found no evidence of VPA or juvenile handling effects on exploration. All mice performed a similar amount of vertical explorations in the self-grooming test [prenatal treatment: F (1, 26.142) = 0.033, p = 0.857; juvenile treatment: F (1, 27.610) = 2.873, p = 0.101; Fig. 2e], walked a similar distance in the open field [prenatal treatment: F (1, 48) = 1.545, p = 0.220; juvenile treatment: F (1, 48) = 0.022, p = 0.884; Fig. 2g] and in the Y maze [prenatal treatment: F (1, 23.065) = 1.638, p = 0.213; juvenile treatment: F (1, 26.740) = 2.764, p = 0.108; Fig. 2i]. In addition, we did not find evidence of anxiety-related behavior in the open field, as all animals travelled a similar percentage of distance in the center [prenatal treatment: F (1, 22.143) = 0.922, p = 0.347; juvenile treatment: F (1, 22.861) = 0.037, p = 0.849, Supplemental Fig. 2]. These data show that neither prenatal VPA exposure nor JH affect adult exploration.

### Prenatal exposure to VPA results in an increase in immobility in the forced swim test (FST), and juvenile handling rescues this behavioral alteration

We then evaluated immobility in the tail suspension test (TST) and FST. Different depression treatments can reduce immobility in these tests^27,28^ and this parameter is commonly used to measure depression-related behavior^29,30^, although some researchers have suggested these tests may actually measure behavioral despair^31^ or a coping strategy to stress^32^.

**Figure 3:**
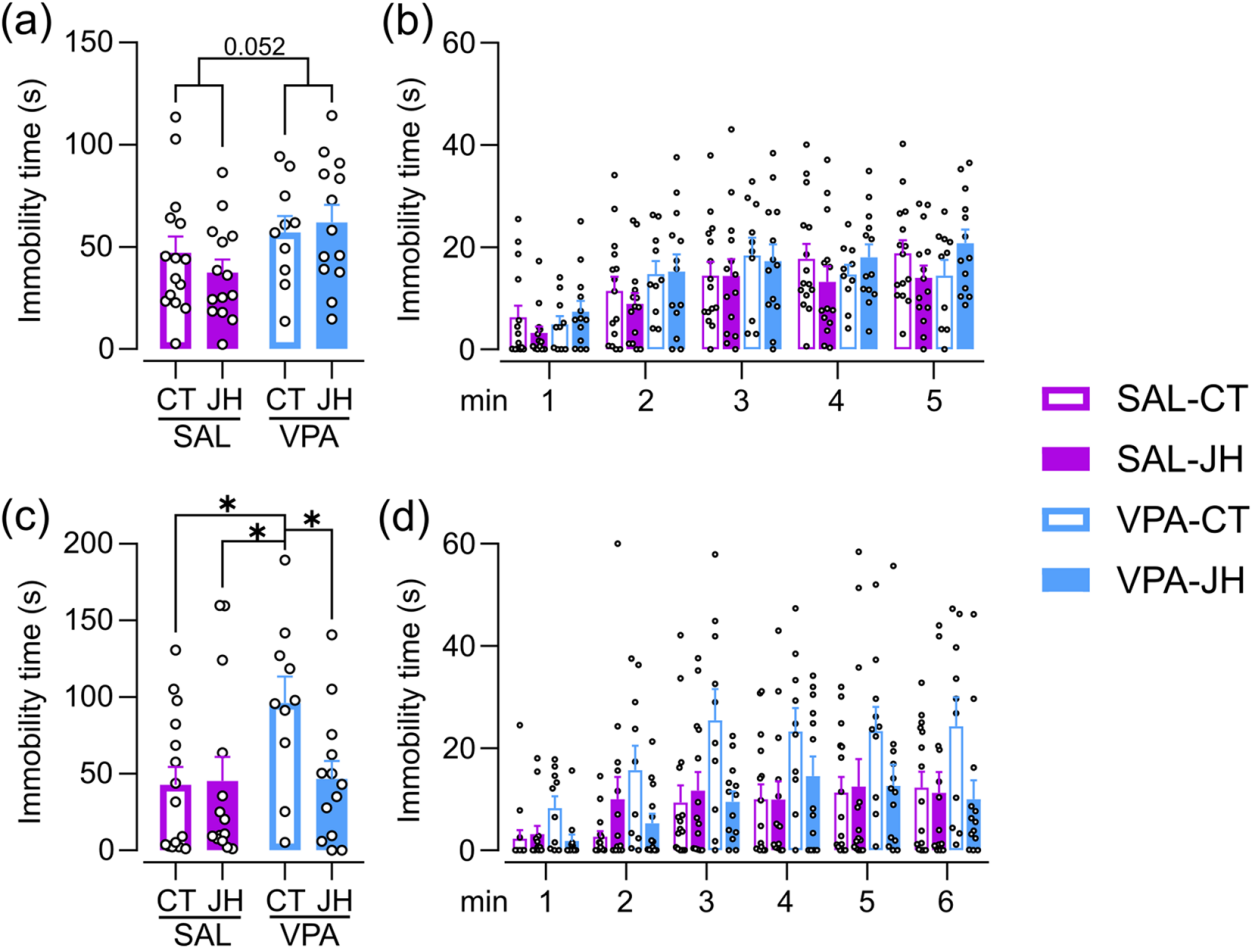
Effect of prenatal VPA exposure and JH on depression-related behaviors. (**a**) VPA-exposed animals showed a tendency to spend more time immobile during the tail suspension test in a LME model, (**b**) with no differences in any minute bin. (**c**) VPA-CT animals spent more time immobile than control and handled groups in the final 3–6 min period of the forced swim test. GLMM model followed by post hoc comparisons with FDR correction, *p < 0.05. (**d**) VPA-CT increased immobility is observed across all minute bins. Data are shown as individual values (dots) and mean + s.e.m. N_SAL-CT_ = 15, N_SAL-JH_ = 14, N_VPA-CT_ = 10, N_VPA-JH_ = 13.

In the TST, we observed a tendency of VPA-exposed animals to spend more time immobile in the total 5-min period [prenatal treatment: F (1, 25.593) = 4.138, p = 0.052; juvenile treatment: F (1, 26.114) = 0.181, p = 0.674; Fig. 3a]. Immobility increased in all groups over time [F (4, 192) = 26.537, p < 0.001; Fig. 3b] and no interaction of prenatal or juvenile treatments with time were found [prenatal treatment × time: F (4, 192) = 1.435, p = 0.223; juvenile treatment × time: F (4, 192) = 0.168, p = 0.954; Fig. 3b].

In the FST, we observed an interaction between treatments in the time spent immobile in the final 3–6 min period [GLMM, χ^2^ (1, 46) = 4.038, p = 0.044], with VPA-CT animals spending more time immobile than the other groups (Fig. 3c). In addition, the analysis over time revealed all mice increased their immobility through the test [χ^2^ (5, 282) = 1775.583, p < 0.001; Fig. 3b] and we found an interaction between prenatal treatment, juvenile treatment and time [χ^2^ (5, 282) = 430.755, p < 0.001]. Remarkably, the effect of JH rescuing the increment in immobility observed in VPA-exposed animals can be observed along all the 1-min bins (Fig. 3d).

### Prenatal VPA exposure results in increased neuronal activity in the piriform cortex, and juvenile handling reverts this effect

**Figure 4:**
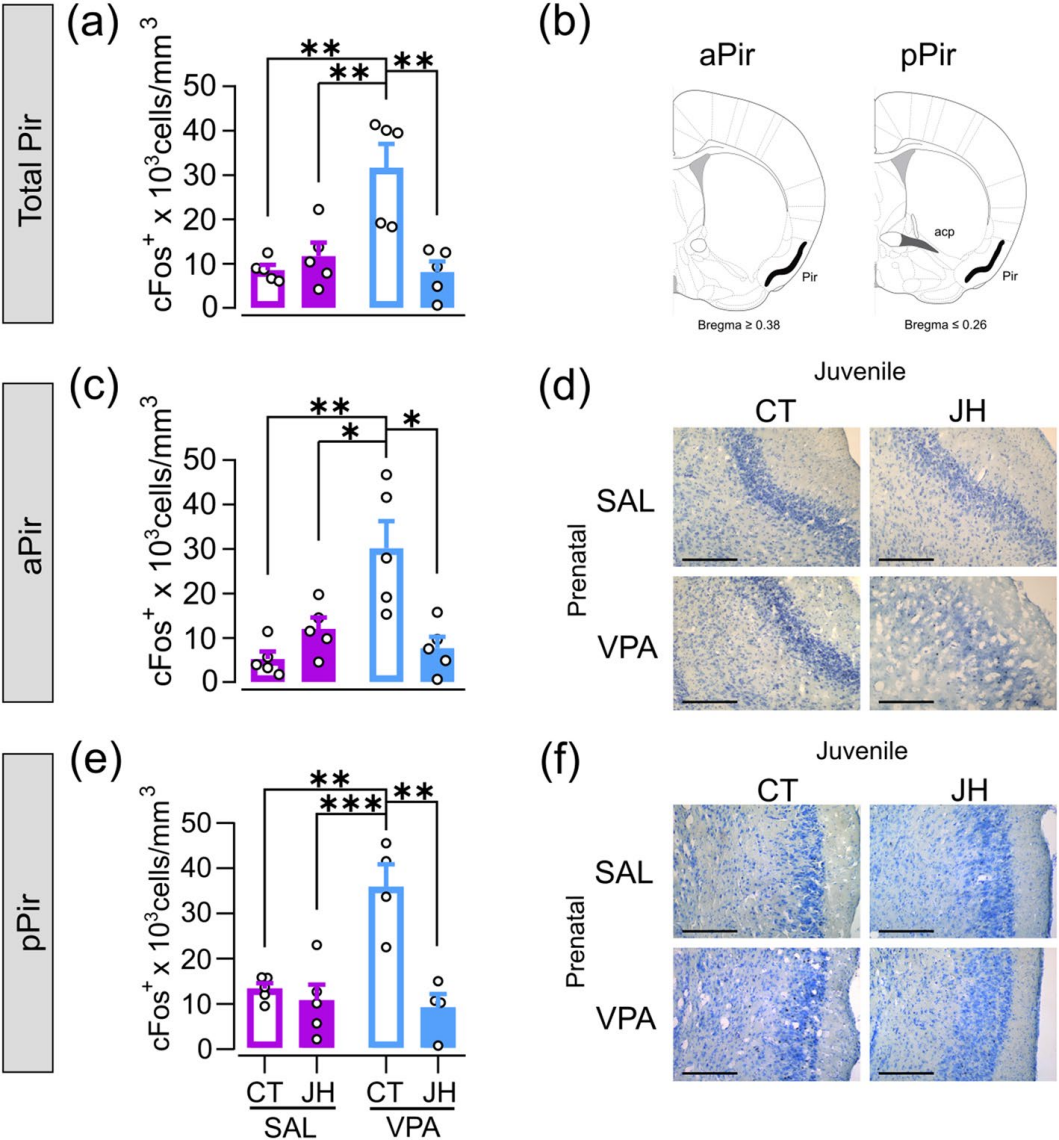
Effect of prenatal VPA and JH on neuronal activity in the piriform cortex (Pir). (**a**) Density of cFos-positive cells in the layer 2 of the Pir is larger in VPA-CT animals when compared with the other groups. (**b**) Scheme showing the limit for the anterior Pir (aPir) and the posterior Pir (pPir). VPA-CT animals show increased cFos-positive cell density in the aPir (**c**) and the pPir (**e**). LME models followed by Tukey’s post hoc comparisons, *p < 0.05. Data are shown as individual values (dots) and mean + s.e.m. N = 4–5 per group. Representative sections subjected to cFos immunohistochemistry and Nissl staining of the aPir (**d**) and pPir (**f**). Bars, 200 μm.

We previously found an increase in glucose metabolism and neuronal activity in the Pir of VPA-exposed mice and demonstrated that early social stimulation is sufficient to revert this phenotype^10^. Neuronal activity can be studied by analyzing the extent of expression of the immediate early gene cFos. We analyzed cFos expression in the Pir in a subset of behaviorally tested animals and found an interaction between prenatal and juvenile treatments on this parameter [F (1, 6.674) = 15.908, p = 0.006]. A post hoc analysis yielded that the layer 2 of the Pir of VPA-CT animals presents an increase in cFos-positive nuclei, a phenomenon that is reversed after handling the mice between PD22 and PD34 (Fig. 4a). To further characterize this effect, we analyzed the anterior (aPir) and posterior (pPir) regions of the Pir (Fig. 4b). We found an interaction between prenatal and juvenile treatments in both the aPir [F (1, 4.914) = 16.137, p = 0.011; Fig. 4c,d] and the pPir [F (1, 8.547) = 14.185, p = 0.005; Fig. 4e,f]. In both cases, post hoc analyses evidenced that the animals of the VPA-CT group have more cFos-positive nuclei in these brain regions than control mice, and this is reversed by the JH procedure.

### Prenatal VPA exposure results in autism-related behavior and this behavior parallels neuronal activity in the Pir

**Figure 5:**
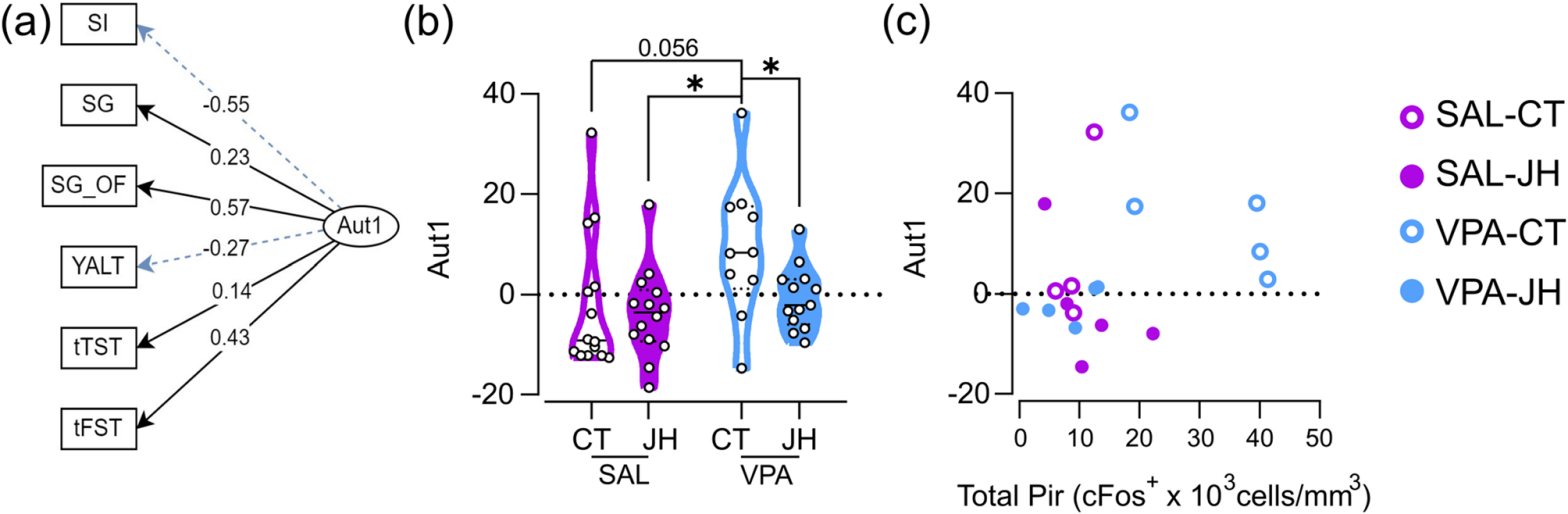
Effect of VPA and JH on an “autism” factor (Aut1). (**a**) Loading values of individual behaviors in the Aut1 factor. *SI* sociability index, *SG* self-grooming time, *SG_OF* time spent grooming in the open field, *YALT* percentage of alternations in the Y-maze, *tTST* time spent immobile in the TST, *tFST* time spent immobile in the FST. Blue, dashed lines indicate negative loadings. (**b**) VPA-CT animals show higher Aut1 scores than all other groups. Data are shown as individual values (dots) and violin plots. LME model followed by Tukey’s post hoc comparisons, *p < 0.05. N_SAL-CT_ = 14, N_SAL-JH_ = 14, N_VPA-CT_ = 10, N_VPA-JH_ = 13. (**c**) VPA-CT animals show high cFos-positive cell density in the Pir and high Aut1 scores, that discriminates them from the other experimental groups. Each animal is represented by a dot. N = 4–5 per group.

To evaluate the behavior of each animal across all tests performed, we carried out a one-factor confirmatory analysis (Aut1) that loads six variables (sociability index, self-grooming time, self-grooming in the open field, Y-maze alternations, immobility time in the TST and immobility time in the FST; Fig. 5a). Statistical analysis revealed significant effects of prenatal and juvenile treatments [prenatal treatment: F (1, 24.629) = 4.389, p = 0.047; juvenile treatment: F (1, 25.188) = 5.780, p = 0.024], with VPA-CT animals having a higher score than SAL-JH and VPA-JH (Fig. 5b).

We expressed Aut1 as a function of cFos-positive cell density in the Pir to evaluate whether Aut1 can predict the level of cFos expression in the Pir (Fig. 5c). Remarkably, VPA-CT animals have high levels of both Aut1 and cFos-positive cell density showing a distribution that differentiates them from all other experimental groups. In addition, VPA-JH values are intermixed with control animals, showing a rescued phenotype in both normal autism-related behaviors and low neuronal activity in the Pir.

## Discussion

Identifying early environmental factors with long-lasting effects on behavior can have profound implications on both basic research and translational neuroscience. Here, we show that a common animal manipulation (handling) performed in a juvenile period (between PD22 and PD34) can affect the expression of repetitive behaviors and rescue the deficit in sociability observed in male mice prenatally exposed to VPA. Our results indicate that care should be taken in experimental designs that include manipulating young animals and testing their behavior as adults. For example, performing behavioral tests in mice around weaning and then again as adults should be correctly controlled, as should designs that involve injecting or weighing mice during the juvenile period. These considerations may be crucial to warrant reproducibility in neuroscience research. In addition, we identified a key time window in which environmental factors have clear effects on disease-relevant behaviors such as those related to ASD. Understanding the cellular and molecular mechanisms that mediate these effects could lead to the uncover of new therapies and treatments.

ASD is usually diagnosed around the age of 2 to 4 years old, when children get involved in social play and interact more actively with peers^1,33^. We have previously shown that social enrichment (i.e. being reared in cages containing animals with normal sociability) can rescue the negative effect of prenatal VPA on mice adult social behaviors^10^. This suggested the existence of a critical developmental period between PD21 and PD60, when sociability can be modulated by environmental factors. Rodents increase their sociability levels between PD21 and PD35, and social isolation during this specific juvenile period can lead to behavioral and functional alterations later in life^34–36^. Therefore, we hypothesized that this is the critical period in which levels of sociability are determined in the VPA model and performed a simple environmental manipulation to test this notion: Handling animals every other day between PD22 and PD34 revealed a positive effect on autism-related behaviors affected by prenatal VPA exposure, supporting the hypothesis.

The term “handling” is employed to refer to different protocols of interaction between a human experimenter and a rodent. Neonatal or early handling involves separating lactating pups from their dam during diverse amounts of time, usually between one to 15 min, and either weigh, manipulate or leave the pups in a box^37–44^. Neonatal handling is usually performed between PD1 and PD21, although shorter protocols are also reported. This experimental approach has been extensively used to study the long-lasting effects of altering the infant environment and showed to affect the outcome of various behaviors, including reduced fear and stress responses, diminished anxiety, increased exploration, decreased sociability and improved learning^38–42,44^. Morphological and molecular changes in the brain that contribute to these behavioral effects have also been identified^45,46^. In addition to the wide variety of protocols employed, neonatal handling involves two stimuli that may have opposite effects: the stress of being separated from the dam on the one hand, and the handling itself on the other hand. These characteristics of the model make its interpretation controversial^43,47^.

In weaned animals, “handling” refers to the habituation of the animal to the manipulation by a human or even to the procedure that involves routine interactions during cage cleaning and breeding. The habituation to handling is usually recommended to reduce the stress caused by the necessary manipulation, particularly in behavioral studies, and it has received special interest in recent years^48,49^. The acute behavioral effects of handling adult animals include improved spatial learning and a mild reduction in anxiety-like behavior^48^, and also reduced aggression^49^. No reports on long-lasting effects on adult behavior after adult handling have been published.

Here we used a subtle handling procedure during the juvenile period and evaluated its long-term consequences. In particular, we found that juvenile handling can rescue the alterations in sociability, repetitive behaviors and depression-related behaviors observed in VPA males. This can also be observed when behavior is analyzed across all tests, in each individual mouse, using the “autism” factor Aut1. In addition, we observed that VPA exposure determines an increment in neuronal activity in the Pir, and juvenile handling reverts this effect. Our results show that handling animals during a juvenile period can have profound effects on adult behavior and brain activity. Handling induces stress in mice, and the reiteration of this procedure can affect the response of the animal to the experimenter and to the behavioral challenge^50^. Even though the human hand bears no superficial resemblance to a rodent, its movements can exert social attraction and may act as social stimulation^51^. Whether our results are due to the chronic mild stress of handling mice, their habituation to manipulation or the activation of mechanisms involving social stimulation, needs to be further studied.

Our observation that juvenile handling rescues the deficit in sociability observed in VPA mice goes in line with our previous report on the effect of early social enrichment^10^ and others showing this recovery after 4 weeks of environmental enrichment from 4 weeks of age^52^. Handling rats neonatally (15 min of separation between PD2 and PD14) results in adolescent animals (PD35) who exhibit reduced neophobia in a social situation and solicitate play more frequently^53^. However, adult handling did not affect the performance of mice in a social interaction test^48^. Similarly, we did not observe an effect of juvenile handling on the sociability of control mice (SAL-JH group). These results may be due to a ceiling effect on social behavior in the three-chamber test. Alternatively, sociability may be determined in the early postnatal period in mice, but VPA could extend this developmental window to the juvenile period, allowing the handling protocol to act upon the maturating neuronal circuits. These alternatives need to be further studied.

We show that the prenatal exposure to VPA results in an increase in the time that mice spent grooming, consistent with previous reports^10^. We also observed that juvenile handling was sufficient to revert the effects caused by prenatal exposure to VPA in the self-grooming test. In addition, in the open field test we observed an effect of juvenile treatment on grooming, as handled animals spent less time performing this behavior. These results show that repetitive behaviors are more sensitive to handling than they are to social enrichment, as this behavior is not rescued in VPA animals reared with control mice^10^ or in BTBR mice reared with C57BL/6J mice^54^. Conversely, BTBR excessive self-grooming is reversed when mice are reared in an enriched environment for 30 days^55^. Self-grooming is an innate behavior related to the mouse hygiene, but it is also elicited by stressful situations and used for social communication^56^. Neonatal handling results in a significant increase in the latency to groom and a decrease in the mean duration of self-grooming bouts in adult rats performing the light–dark, holeboard or hyponeophagia tests^57^. Both neonatal and juvenile handling may be activating neuronal circuits key to this repetitive behavior and affecting its developmental trajectory.

We also observed that juvenile handling was sufficient to revert the alterations in the immobility time observed in VPA animals in the FST. Strikingly, we did not find significant differences between groups in the TST. Although TST and FST are conceptually related since both render a situation in which the animal alternates between trying to escape the stressful environment and choosing to remain immobile^27,28^, they are probably different in terms of the biological substrates that underlie the observed behavior^29^. On the one hand, TST is more affected by variability between individual mice and protocols recommend 15 mice per group, while 10 mice per group should suffice in the FST^58^. Therefore, our lack of effects on the TST may be related to the number of animals used in this test (10–15 mice/group). On the other hand, both tests differ in their differential sensitivity to the immobility-reducing effects of various antidepressants^29^, and similarly JH may affect one but not the other. Worth to mention here, neonatal handling (15 min separation from PD1 to PD22) did not affect the performance of Wistar rats in the FST^59^, adult handling did not affect mice performance on neither the FST nor the TST^48^, and social enrichment was also unable to reverse VPA increment in immobility in the FST^10^, underscoring that different effects on these behaviors are observed after similar environmental manipulations. The mechanisms by which juvenile handling reduces the time that VPA mice spend immobile in the FST need to be further studied.

Social behavior is regulated by various brain structures that are altered in ASD: the fusiform face area (mediates the perception of personal identity), the inferior frontal gyrus (which allows to imitate facial expressions), the posterior superior temporal sulcus (responsible for the perception of facial expressions and involved in eye gaze tasks), the superior frontal gyrus (neuronal substrate of the theory of mind) and the amygdala (involved in emotion processing)^60^. In addition to the mentioned areas comprising the social brain, other areas are reported to be involved in the alterations observed in ASD. In particular, ASD is often characterized by atypical sensory behavior, showing for example decreased olfactory discrimination scores^61^. These alterations could be explained by the dystrophic serotonin axons reported in the Pir of ASD individuals^62^. In rodents the structures underlying social behaviors are largely related to the olfactory system. For example, there is a significant induction of cFos expression in the Pir of mice after exposure to a social encounter^63^ and social learning is regulated by the hypothalamic neuropeptide oxytocin acting on neurons in the Pir^64^. Here we repeat a previous observation of increased cFos expression in the Pir of VPA mice^10^ and show that this parameter is reversed in VPA-JH mice. The effect of juvenile handling on other alterations previously observed in the Pir of VPA-exposed animals, such as hypomyelination and alterations in oligodendrocyte-lineage cells^65^, needs to be evaluated.

The loading of key autism-related behaviors in a factor that has high values in VPA-CT animals, and for which VPA-JH mice show low values similar to control mice, supports not only the validity of the model but also the ability of juvenile handling to have a positive effect across multiple domains of behavior. Such a score can be very valuable to assess, for example, candidate cellular and molecular alterations in the VPA model. As an example, we studied the pattern of the increased density of cFos-positive cells in the Pir in our experimental groups. While no individual behavior could discriminate between aberrant vs normal Pir neuronal activity, high Aut1 values allowed for a good separation of these animals. Interestingly, VPA mice that were handled between PD22 and PD34 were indistinguishable from control animals in both Aut1 scores and cFos expression, showing that the juvenile treatment can fully rescue animals from the prenatal VPA effects. We propose the use of composite factors to help identify other cellular alterations that underlie the complex abnormal behavior observed in animal models of psychiatric disorders.

In summary, our results first bring attention to the design of experimental protocols, due to the long-term consequences that simple manipulations such as handling the animals as juveniles can have on adult behavior. Second, we identified a juvenile developmental window of susceptibility, when environmental factors can affect adult behavior, adding to our previous report of social enrichment. Finally, we particularly show that juvenile handling can rescue the detrimental effects of prenatal VPA exposure on sociability, repetitive behaviors, and depression-related behavior, and underscore the possible role of the Pir in these behaviors.

## Materials and methods

### Animals and handling procedure

Outbred CrlFcen:CF1 female and male adult mice were obtained from the animal house of the Faculty of Exact and Natural Sciences, University of Buenos Aires (Buenos Aires, Argentina). All animals were housed on a 12:12 light/dark cycle with lights on at 6 a.m., constant room temperature (18–22 °C), and food and water ad libitum. All animal procedures were performed according to the regulations for the use of laboratory animals of the National Institute of Health (Washington, DC, USA) and approved by the institutional animal care and use committee of the Faculty of Exact and Natural Sciences, University of Buenos Aires (CICUAL Protocol Nr. 128b).

The experimental outline is presented in Fig. 1a. Eight-week-old males were mated with eight-week-old nulliparous females and, when vaginal plug was observed, the female was returned to the cage and the day marked as gestational day (GD) 0.5. On GD12.5, pregnant females were injected subcutaneously either with 600 mg/kg of VPA in 0.9% NaCl (Sigma, St. Louis, MO, USA) or with vehicle (SAL), and housed individually.

The day of birth was considered as postnatal day (PD) 0. Litters were culled to ten pups to minimize litter size effects. At PD21, all pups were weaned in cages containing three to five animals, all belonging to the same prenatal treatment (SAL or VPA). Littermates were weaned in different cages. Then, postnatal treatments were randomly assigned to cages: juvenile handling (JH) or control (CT). The design involved four experimental groups: SAL-CT, SAL-JH, VPA-CT, and VPA-JH. Since we previously observed that VPA produces alterations in sociability in males but not in females^17^, only males were used in these experiments.

JH animals were handled for 3 min every other day from PD22 to PD34. This handling procedure was modified from Ref.^44^. Briefly, the investigator (A.S.) took each JH animal from the cage and, wearing thin, latex gloves, gently handled each mouse for 3 min, using both hands. After handling, mice were returned to their homecages. CT animals were left undisturbed in their cages.

Sample size was decided from previous works on the VPA model in this mouse strain and behavioral analyses^10,66^. To control for the litter effect^67^, only one or two animals from each litter were assigned to each juvenile treatment group and the mother was treated as a random factor in the linear fixed-effects models. Two cohorts were used in these experiments. Cohort 1 consisted of 16 litters (8 SAL and 8 VPA), from which up to four males (2 JH and 2 CT) were randomly selected from each litter and 8 SAL-CT, 8 SAL-JH, 7 VPA-CT and 10 VPA-JH were tested as adults. Cohort 2 consisted of 11 litters (7 SAL and 4 VPA), from which up to two males (1 JH and 1 CT) were randomly selected from each litter, and 7 SAL-CT, 7 SAL-JH, 3 VPA-CT and 4 VPA-JH were tested as adults. Five animals from each treatment (Cohort 1) were used for the quantification of cFos-positive cells.

For experimental design and preparation of the manuscript, we followed the recommendations in the ARRIVE guidelines.

### Adult behavioral testing

All behavioral procedures were carried out during the light period (from 7 a.m. to 5 p.m.), except for the Y-maze test that was performed between 4 p.m. and 8 p.m. to maximize exploration. Tests were carried out in the order listed below, with 1-week interval to minimize the interference between tests (Fig. 1b). Cages and animals within a cage were randomly selected for each test; cages and videos were coded for blind evaluation of behavior. Animals were habituated to the testing room before each test in their home cages for 30 min. In all cases, animals were recorded during the test using a webcam (Logitech Webcam Pro 9000), and their behavior analyzed with the aid of the video-tracking software AnyMAZE (Stoelting, CO, USA).

#### Social interaction test

We followed the protocol previously described^68^. Briefly, a black PVC maze (40.6 cm long × 15 cm wide × 23 cm tall), with three 15 cm × 15 cm chambers covered with clean soil bedding was used. Two identical transparent Plexiglas cylinders (each 7.5 cm in diameter, 15 cm tall) with a plastic Petri dish on top were placed in the maze, one on each lateral chamber. The test was performed under dim light (10 lx) and consists of two phases. During the 5 min habituation phase, both cylinders were empty, and the animal was allowed to freely explore the apparatus. Then, a stimulus mouse (21–35 days old male CF1 mouse) was placed into the cylinder of the aleatorily designated “social chamber”. The opposite chamber was designated as “nonsocial chamber” and an object with similar size and color to the stimulus mouse was placed in its cylinder. Animals were allowed to explore during a 10-min social phase. The cylinders had 0.5 cm diameter holes that allow for auditory, visual, and olfactory investigation between test and stimulus mice. Sniffing time (time spent with the nose in one of the small holes of either of the cylinders) was quantified offline by a trained experimenter (A.S.) blinded to group assignments. The social index was calculated as (time sniffing the social tube − time sniffing the nonsocial tube)/(total exploration time). One animal (SAL-CT) was removed from this test because it was jumping on the walls of the maze the whole time during the test.

#### Self-grooming test

Mice were evaluated for spontaneous grooming as previously described^10^. We used a Plexiglas cylinder (5.5 cm diameter × 20 cm tall) covered with a chinstrap. This test was carried out under dim light (10 lx). Mice were habituated to the cylinder during an hour for two days and, on the third day, each animal was placed in the cylinder and, after 10 min of habituation, its behavior was recorded during 10 min. Grooming time and rearing events were quantified offline by a trained experimenter (A.S.) blinded to group assignments.

#### Open field test

The test was performed as previously described^10^. A black arena was used (45 cm × 45 cm × 30 cm high) under bright illumination (100 lx). A central square (23 cm × 23 cm) was designed as the central area. Each animal was placed near the wall pointing its head to the center, and mice were allowed to freely explore the arena during 15 min. Total locomotion and percentage of time in the center of the field were automatically scored by the software. Grooming time was quantified offline by a trained experimenter (A.S.) blinded to group assignments.

#### Y-maze test

The Y-maze test was performed as previously described^10^. An apparatus with three identical, 42-cm long arms was used. Each animal was placed in the tip of one arm that was randomly assigned as the starting point, and then the animal was allowed to freely explore the three arms during 10 min, under dim light (10 lx). The total distance travelled, and the sequence of arm visits were automatically calculated by the software. Percentage of alternation was calculated as (alternations × 100)/(Total arm visits − 2), where an alternation was considered every time that the mouse consecutively visited the three arms.

#### Tail suspension test (TST)

We followed the protocol previously reported^66^. Animals were suspended by their tails (about 4/5 from the base) to a wire suspended 25 cm above the floor, during 5 min under an illumination of 50 lx. Total immobility time was measured offline by a researcher (A.S.) blinded to treatments. Mice were considered immobile when they hung passively, making no movement.

#### Forced swim test (FST)

This test was performed as previously described^66^. Each animal was gently placed into a glass beaker (27 cm high × 18 cm diameter) filled with 15 cm of warm water (24 °C) under an illumination of 50 lx during 6 min. At the end, animals were dried with a paper towel. Immobility time was quantified by a trained experimenter (A.S.) blinded to group assignments. Mice were considered immobile when they made no movements other than those necessary to balance the body and keep the head above the water^69^.

### cFos immunohistochemistry

Five animals from each treatment were randomly chosen from the cohort 1. Due to processing 2 weeks after the last behavioral test, mice were deeply anesthetized (i.p. 80 mg/kg ketamine chlorhydrate and 8 mg/kg xylazine) and transcardially perfused with heparinized saline followed by 4% paraformaldehyde (PFA) in 0.1 M phosphate buffer (PB), pH 7.2. Brains were post-fixed for 24 h and cryopreserved in 30% sucrose solution in 0.1 M PB at 4 °C. All brains were wrapped in aluminum foil and frozen at − 80 °C in a freezer. 40-µm coronal sections across the Pir were obtained with a cryostat (Peetlab, RD2230) and then stored in cryopreservation solution at − 20 °C. cFos expression analysis was performed as previously described^10^. Briefly, sections were incubated with the primary antibody rabbit anti-cFos (1:1000 in blocking solution; EMD Millipore, Burlington, MA, USA), the secondary antibody biotin-SP-conjugated donkey anti-rabbit (1:200 in blocking solution; Jackson ImmunoResearch, West Grove, PA, USA) and the ABC kit (Vector Laboratories, Burlingame, CA, USA). Sections were then stained with cresyl violet (5 mg/ml in 0.6% acetic acid). Images were obtained using an Infinity1 camera (Lumera Corporation, Ottawa, ON, Canada) and a light-field microscope (Olympus CX31, Buenos Aires, Argentina) at a 400X magnification. Total cFos-positive nuclei were counted in the layer 2 of the Pir and then normalized by the volume of the layer. All measurements were performed with the aid of the Fiji software^70^. For one VPA-CT and one VPA-JH animal, we failed to obtain three pPir sections for quantification, and these animals were not included in the pPir analysis.

### Statistics

We used R^71^ with lmerTest^72^ to fit linear mixed-effects (LME) models for each variable with the mother as a random factor, followed by Tukey’s post hoc comparisons performed with the aid of the emmeans package^73^. In case the data did not meet the assumption of normality and homogeneity of variances, we fit generalized linear mixed-effects (GLMM) models with Poisson and negative binomial distributions with glmmTMB^74^. We performed paired Student’s t tests to assess the preference for each side in the habituation phase and the preference for the social side in the social interaction test. In all cases, statistical significance was assumed where p < 0.05.

To create an “autism” factor, we chose to include six variables that best characterized autism-related behaviors in the tests performed: sociability index (SI) from the social interaction test, self-grooming time (SG) from the self-grooming test, time spent self-grooming in the open field (SG_OF), percentage of alternations in the Y maze (YALT), time spent immobile in the tail suspension test (tTST) and time spent immobile in the final 3–6 min period in the forced swim test (tFST). Prior to the exploratory factor analysis (EFA), we implemented the Kaiser–Meyer–Olkin test and obtained a MSA of 0.6. We then performed the Bartlett’s test of sphericity: χ^2^ = 16.014, df = 15, p = 0.381. Both tests confirmed that the available data was suitable for a dimension reduction. Results of EFA suggested all variables were adequately expressed in one factor. Based on these results, we performed a confirmatory factor analysis (CFA) with the aid of the lavaan package^75^. The global fit measures suggested that the CFA model fits well to the data (χ^2^ = 3.455, df = 9, p = 0.943). Finally, we estimated linear latent scores for each observation and fitted a LME model with the mother as a random factor.

## Supplementary Information

Supplementary Figures.

## Data Availability

All relevant data are presented within the manuscript and available as raw data and R scripts of statistical analyses in https://osf.io/3zbvf/?view_only=34d9176268594c3caf43293555760cf0.
