# Supplementary material for: Juvenile handling rescues autism-related effects of prenatal exposure to valproic acid
Source: Sci Rep. 2022 May 3;12:7174. doi: 10.1038/s41598-022-11269-7 (PMC9065111; doi:10.1038/s41598-022-11269-7)
Supplement: Supplementary file 1 — Supplementary Figures. [file 41598_2022_11269_MOESM1_ESM.docx]

**SUPPLEMENTAL INFORMATION**

**SUPPLEMENTAL FIGURES**


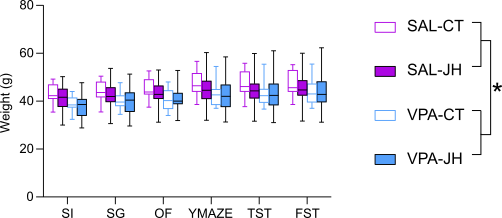


***Supplemental figure 1*. Effect of prenatal VPA exposure ant juvenile handling (JH) on adult body weight.** Animals were weighted after each behavioural test. In comparison to SAL animals, VPA-exposed mice show reduced body weight in adulthood. LME model with repeated measures, * p < 0.05. N_SAL-CT_ = 15, N_SAL-JH_ = 15, N_VPA-CT_ = 10, N_VPA-JH_ =13. Data are shown as boxplot with min-to-max whiskers.

***Supplemental figure 2.* Percentage of distance walked in the center of the open field.** We did not find evidence of anxiety-related behavior in the OF, as all animals walked a similar percentage of their distance in the center. Data are shown as individual values (dots) and mean + s.e.m.
